# Systemic Inflammation in Severe Obese Patients Undergoing Surgery for Obesity and Weight-Related Diseases

**DOI:** 10.1007/s11695-017-3104-9

**Published:** 2018-03-01

**Authors:** Wilson R. Freitas, Luis Vicente Franco Oliveira, Eduardo A. Perez, Elias J. Ilias, Carina P. Lottenberg, Anderson S. Silva, Jessica J. Urbano, Manoel C. Oliveira, Rodolfo P. Vieira, Marcelo Ribeiro-Alves, Vera L. S. Alves, Paulo Kassab, Fabio R. Thuler, Carlos A. Malheiros

**Affiliations:** 1 Master’s Degree and PhD Post Graduation Program in Research in Surgery, Santa Casa of Sao Paulo Medical School, Sao Paulo, SP Brazil; 2 School of Medicine, University Center of Anapolis (UniEvangélica), Rua Graciano A. de Souza 514, Lote 28, Quadra 07, Setor Bougainville, Anapolis, GO 75075-580 Brazil; 3 School of Health Sciences, Santa Casa de Sao Paulo, Sao Paulo, SP Brazil; 4 Sleep Laboratory, Master’s Degree and PhD Programs in Rehabilitation Sciences, Nove de Julho University (UNINOVE), Sao Paulo, SP Brazil; 5 Immunology and Pulmonary Exercise Laboratory, Master’s Degree and PhD Program in Rehabilitation Sciences, Nove de Julho University (UNINOVE), Sao Paulo, SP Brazil; 6 Universidade Brasil, Brazilian Institute of Teaching and Research in Pulmonary and Exercise Immunology (IBEPIPE), Sao Jose dos Campos, SP Brazil; 7 Evandro Chagas Research Institute, Oswaldo Cruz Foundation, Rio de Janeiro, Brazil

**Keywords:** Severe obesity, Bariatric surgical procedure, Surgery for obesity and weight-related diseases, Inflammation, Adipokines, Weight loss, Comorbidity

## Abstract

**Background:**

Obesity is a worldwide disease related to genetic, environmental, and behavioral factors, and it is associated with high rates of morbidity and mortality. Recently, obesity has been characterized by a low-grade inflammatory state known as inflammome indicated by chronic increases in circulating concentrations of inflammatory markers. The purpose of this study was to evaluate the effect of weight loss induced by surgery for obesity and weight-related diseases on pro-inflammatory cytokine (TNF-α) and anti-inflammatory adipokine (adiponectin) levels, and on an adipose-derived hormone (leptin) in severely obese subjects.

**Methods:**

This randomized, controlled trial involved 55 severe obese patients (50 women, age 18–63 years, and body mass index of 35.7–63 kg/m^2^) who underwent bariatric surgery (BS). Patients with a BMI > 65 kg/m^2^ and clinical and mental instability, or significant and unrealistic expectations of surgery were excluded. Blood samples were collected during the fasting period to analyze tumor necrosis factor alpha (TNF-α), adiponectin, and leptin levels by enzyme-linked immunosorbent assay.

**Results:**

At baseline, no significant difference was observed in the anthropometric, demographic, clinical characteristics and biochemistry and inflammatory markers between the control group (CG) and bariatric surgery group (BSG). The same finding was also observed when we compared the baseline variables to those at the 6-month follow-up in the CG. However, the same variables in the BSG group were significantly different between baseline and the 6-month follow-up after BS.

**Conclusions:**

Weight loss induced by surgery for obesity and weight-related diseases reduced the inflammome state in severely obese patients.

## Introduction/Purpose

The prevalence of obesity is growing worldwide and has become a major global health challenge. Both overweight and obesity are characterized by the accumulation of excessive levels of body fat, and this creates an increased risk of cardiovascular diseases, some types of cancer, and overall mortality [1, 2]. Obesity is a worldwide disease related to genetic, environmental, and behavioral factors, and it is associated with high rates of morbidity and mortality. The prevalence of obesity is increasing worldwide, and it has become a main global health challenge [3].

Currently, overweight and obesity are classified by body mass index (BMI). In adults, overweight is defined as a BMI (weight in kilograms/height^2^ in meters) of 25.0 to 29.9 kg/m^2^, whereas obesity is defined as a BMI ≥ 30.0 kg/m^2^ [2]. Both overweight and obesity are characterized by the accumulation of excessive levels of body fat, and this creates an increased risk of cardiovascular diseases (CVD), type 2 diabetes mellitus (T2D), hypertension, stroke, certain types of cancer, gallbladder disease, dyslipidemia, osteoarthritis and gout, pulmonary diseases, sleep apnea, and several other associated pathologies [4, 5]. In particular, abdominal fat, which is metabolically active, is associated with low-grade systemic inflammation and immune activation [5]. Enlarged adipocytes and activated macrophages secrete pro-inflammatory cytokines, such as interferon-c, and hormones, such as leptin, which favor the cell-mediated Th-1-type immune response [2, 6].

According to Flegal et al., the prevalence of obesity has increased dramatically in the last three decades, generating a worldwide critical situation in the management of public health. In the USA, 35% of the adult population is obese (BMI ≥ 30 kg/m^2^), and an additional 35% of the population is overweight (BMI 25 to 29.9 kg/m^2^) [2].

The world prevalence of obesity more than doubled between 1980 and 2014. The World Health Organization reported that by the end of 2014, more than 1.9 billion adults were overweight (38% of men and 40% of women), and more than 600 million were obese (11% of men and 15% of women) [1]. Still, according to these data, in 2013, 42 million children less than 5 years old were overweight or obese. In emerging countries, the increase in overweight and childhood obesity was greater than 30%, which was higher than that in developed countries. In Brazil, 16.8% of men and 24.4% of women were obese, while 56% of the adult population was overweight in 2013 [7]. Therefore, Brazil emerges as the second country in the world, behind only the USA, to have a high number of bariatric surgery (BS), with more than 95,000 operations performed per year. In the last 10 years, the number of BS increased by 300%, and the risk of this procedure is currently equivalent to a medium-sized abdominal surgery [8].

Presently, obesity is recognized as a chronic progressive disease, which has already reached pandemic proportions, and it is considered one of the leading causes of death and disability worldwide [3]. It severely compromises children and adults, causing a high number of correlated diseases, including most non-communicable diseases [4, 5].

The treatment of obesity and its associated health problems represents a significant economic impact on health systems, as the prevalence of obesity increases at an alarming rate. The treatment of obesity in the long term is very unsatisfactory because of its complex pathophysiology and the difficulties inherent to patients maintaining modifications to their lifestyle [9, 10].

Although adipose tissue is no longer considered an inert tissue mainly devoted to energy storage, it is emerging as an active participant in regulating physiologic and pathologic processes, including immunity and inflammation [11]. White adipose tissue plays an important role in the storage of lipids, and it has outstanding endocrine function, as it secretes various hormones, including leptin and adiponectin, as well as various other protein factors [12]. Fat cells secrete adipokine, which together with fatty acids and prostaglandins are involved in lipid metabolism, insulin sensitivity, the alternative complement system, vascular hemostasis, blood pressure regulation and angiogenesis, and energy balance regulation [12].

In addition, numerous adipokines are involved in the inflammatory process (TNF-α, interleukin [IL]-1b, IL-6, IL-8, IL-10, transforming growth factor b, and nerve growth factor) and acute phase response (plasminogen activator inhibitor-1, haptoglobin, and serum amyloid A) [13–17]. Physicians should also consider that circulating concentrations of plasminogen activator inhibitor-1, angiotensin II, C-reactive protein, fibrinogen, and TNF-α are all related to BMI [18, 19]. In overweight and obesity, the production of these proteins by adipose tissue is increased; consequently, the high circulating levels of inflammatory cytokines characterize a chronic low-grade inflammation state with a direct causal statement of insulin resistance and metabolic syndrome [12]. This concept is very important in understanding obesity as a health condition. Obesity plays an important role in the pathophysiology of various diseases through the complex interaction among dietary, genetic, and metabolic factors. This chronic metabolic state of a low level of inflammation, inflammome, is caused and maintained by the relationship between adipose tissue and the metabolisms of lipids and glucose. Therefore, obesity should be understood as the basis of many diseases and not just as excess adipose tissue [20].

Thus, obesity or excess body fat has become better understood since 2005. Currently, adipose tissue is considered as highly secretory tissue, metabolically active, and responsive to appetite modulators, energy imbalances, insulin resistance, reproductive and endocrinological systems, bone metabolism, immunity, and inflammation [5, 11].

According to the Position Statement of the International Federation for the Surgery of Obesity and Metabolic Disorders (2016) on the Indications for Surgery for Obesity and Related-Related Diseases, a comprehensive, proactive strategy is urgently needed to deal with the challenges facing the current global obesity epidemic. The actions of obesity prevention in public health should be the most aggressive and a priority. However, considering the excessive number of obese patients, surgery is presently the most effective treatment and the only long-lasting option for this population [9].

Surgical and endoscopic options should be considered and offered to individuals with obesity and weight-related diseases. Surgery for obesity and weight-related diseases has been proven to be highly efficacious in treating obesity and its comorbidities. Currently, surgery for obesity and weight-related diseases resolves more than 75% of morbid obesity and super obesity and is equally effective for weight-related comorbidities and complications [9].

BS should be considered when other non-surgical therapeutic approaches, such as lifestyle modifications and pharmacological therapies, have been ineffective. Surgery improves outcomes in relation to long-term weight loss and associated comorbidities compared to non-surgical interventions regardless of the surgical technique used [21, 22].

BS results in significant and sustained weight loss in morbidly obese subjects with minor morbidity or mortality. Some studies have shown that systemic inflammation in obese subjects seems considerably reduced after BS [23–26].

Arismendi et al. have confirmed and expanded on previous studies’ results by providing an integrated network approach of the interplay among the different inflammatory markers (inflammome) [27]. According to these authors, morbid obesity is associated with a significant systemic inflammome that is not influenced by sex, smoking status, the presence of obstructive sleep apnea, and/or metabolic syndrome, and it is considerably ameliorated after BS. Therefore, the purpose of this study was to evaluate the effect of weight loss induced by BS on pro-inflammatory cytokine (TNF-α) and anti-inflammatory adipokine (adiponectin) levels, and on an adipose-derived hormone (leptin) in severely obese patients.

## Materials and Methods

### Trial Design

**Fig. 1:**
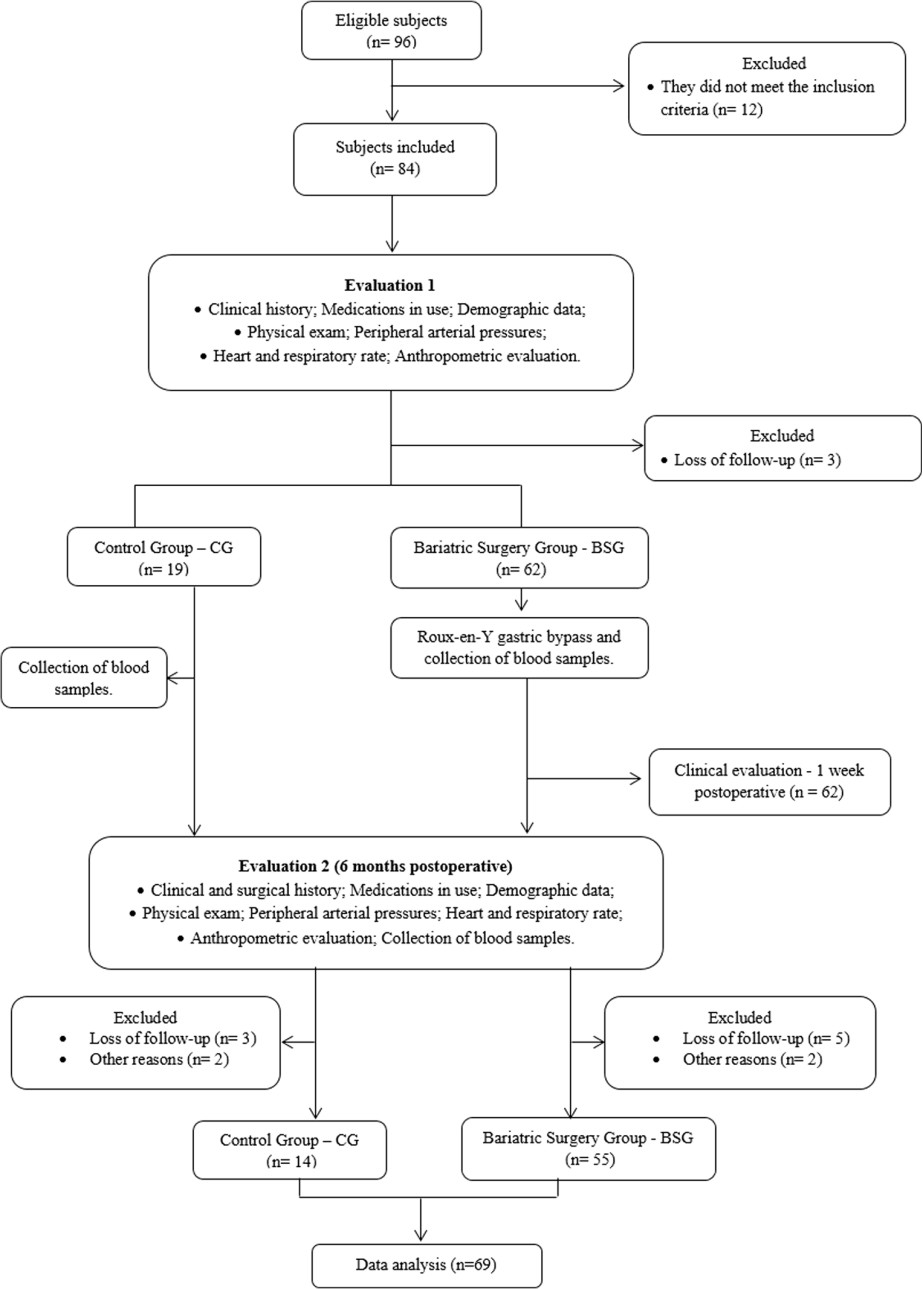
Flow diagram of the study

This randomized, controlled clinical trial was conducted, reviewed, analyzed, and reported according to the SPIRIT (Standard Protocol Items: Recommendations for Interventional Trials), and according to the international ethical standards [28]. Figure 1 summarizes the study’s flow diagram.

### Study Setting

This study was performed at the Santa Casa Medical School (São Paulo, Brazil) and Immunology and Pulmonary Exercise Laboratory of Nove de Julho University (Sao Paulo, SP, Brazil). Physicians and surgeons of the Department of Gastric Surgery at Santa Casa Medical School (Sao Paulo, Brazil) performed all surgical procedures. Patients were examined preoperatively, intraoperatively, and at 6 months after BS.

### Subjects

#### Eligibility Criteria

The following inclusion criteria were required for randomization: male and female patients; those aged 18–65 years; patients with grade III obesity (BMI ≥ 40 kg/m^2^ or ≥ 35 kg/m^2^ with comorbidities); those awaiting BS; patients with a documented history of failed conventional weight loss attempts; and those with the ability to understand and agree to participate in the study, and sign an informed consent form. Exclusion criteria were as follows: patients with a BMI > 65 kg/m^2^; those with an unrealistic postoperative target weight and/or unrealistic expectations regarding surgical treatment; patients who were pregnant, lactating, or had a planned pregnancy within 2 years; those in whom safe access to the abdominal cavity or gastrointestinal tract was lacking; patients with alcohol abuse or drug use; those with cancer; patients with a previous diagnosis of autoimmune disease; and those with any cardiorespiratory and/or medical condition that contraindicates surgery.

#### Recruitment and Randomization

We prospectively recruited clinically stable, severely obese patients from the Bariatric Surgery Outpatient Clinic (Santa Casa Medical School), and randomized eligible patients in a 3:1 proportion between the bariatric surgery group (BSG) and control group (CG). Randomization numbers were automatically generated using a computer and randomization table at a central office. Generated numbers were placed in opaque, sealed envelopes to ensure allocation blinding, and each envelope contained a card number that indicated the patient’s allocation. This randomization method was used because of the large number of patients on the waiting list for BS and the limited ability to meet the demand for surgical procedures at our public hospital. Patients in the CG returned to the waiting list after the 180-day study period, or if they presented with any clinical complications indicating urgent BS.

### Pre-surgical Procedures

Obese individuals and healthy controls were interviewed in detail regarding their family history of obesity, eating habits, medication use, and physical activity; data were recorded in a form specifically designed for this purpose. All patients underwent a medical examination and pre-operative assessment. Additionally, anthropometric measurements were taken, and fasting blood samples were collected at baseline and 6 months postoperatively.

### Body Parameters

Anthropometric measurements with an emphasis on clinical markers of adiposity were obtained before breakfast, with patients wearing light clothing without shoes. The BMI was calculated as weight (kg) divided by height squared (m^2^). The waist circumference was measured using a soft tape in the standing position following normal expiration; waist was defined as the narrowest circumference between the costal margin and iliac crest. The neck circumference was measured by taking the circumference of the cricothyroid membrane and superior border of the seventh cervical spinous process [3, 29].

### Blood Biochemical Analysis

Venous blood samples (5 mL) were obtained in the morning after 12 h of fasting by puncturing the cubital vein and collected in vacuum tubes (Vacuette do Brasil Ltda, Campinas, São Paulo, Brazil) with a serum clot activator or ethylenediaminetetraacetic acid (EDTA) for plasma preparation. For protein analyses, venous blood was sampled into EDTA tubes and kept on ice until they were centrifuged at 4 °C for 10 min at 2000 g. Plasma aliquots were stored at −80 °C until analyzed. Samples were extracted to a blood cell count using a hematology analyzer (XS-800i; Sysmex, Roche, CA, USA) to quantify total cholesterol (TC), high-density lipoprotein (HDL) cholesterol, low-density lipoprotein (LDL) cholesterol, triglyceride, and glucose using commercial kits (Gold Analisa Diagnóstica Ltda, Belo Horizonte, MG, Brazil) and the SpectraMax i3 (Molecular Devices, Sunnyvale, CA, USA). The TNF-α, leptin, and adiponectin levels were analyzed by enzyme-linked immunosorbent assay kits from R&D Systems (Minneapolis, MN, USA) and Biolegend (Sellex Inc., Washington, DC, USA), according to the manufacturers’ recommendations. Spectrophotometric readings were performed using a SpectraMax i3x Multi-Mode Detection Platform (Molecular Devices, LLC, Sunnyvale, CA, USA). The blood sample collection and analysis of the CG was held during the baseline assessment and follow-up, approximately 6 to 8 months, according to the same protocol.

### Surgical Procedures

All patients were operated on by three surgeons who alternated between the surgeon and two assistant in each surgery. Informed consent for the surgery and research study was obtained from all patients.

### Surgical Technique

Patients received general anesthesia and then the first blood sample was collected. Patients were placed in the horizontal dorsal decubitus position with a sequential compression device for deep vein thrombosis prophylaxis. Skin prepping using chlorhexidine and draping were performed in the usual standard surgical manner. The abdominal incision was marked and started from 2 cm below the xiphoid process to 7 cm above the umbilicus. Surgical procedures were of the gastric bypass type with Roux-en-Y reconstruction, with a small pouch kind of Capella with gastrointestinal anastomosis in two sutures, being one of continuous 4-0 Vicryl and the other of seromuscular cotton 3-0 with sutures, with lateral anastomosis (1.5 cm in diameter). No silastic ring was placed. The loop food was 100 cm, and the handle was 70 cm biliopancreatic with enteroanastomosis lateral side 3-0 Vicryl running suture in two layers with a diameter of 4 cm. This was first described in 2014 [30].

### Post-Surgical Procedures

No patients stayed in the intensive care unit during the postoperative period. All patients were transferred to the surgical ward. Patients were allowed to take sips of water on the first postoperative day and were usually discharged on the third postoperative day. Patients were advised that they should continue drinking 30 mL of clear fluids every 1 and a half hour for 2 weeks until their first postoperative follow-up visit. Patients were instructed to undergo their first follow-up visit in the outpatient clinic 2 weeks postoperatively, during which wound care was performed. If there were no complaints, patients were allowed to start a soft diet for another 2 weeks and then eat small, non-sweet, frequent meals from the fifth postoperative week. Patients were followed up with at 3, 6, and 12 months postoperatively. During the follow-up at 6 months, patients’ weight was measured, and blood samples were taken to compare postoperative biochemical and metabolic variables with the baseline ones.

### Outcomes

The primary outcome was the change in systemic inflammatory status 6 months after the BS, as determined by inflammatory markers in the fasting blood samples.

### Statistical Analysis

Numerical data are presented as a mean and standard deviation in the case of variables with a normal and median distribution or interquartile range for variables with an asymmetric distribution. Categorical data are described in absolute numbers and percentages of the total. The Kolmogorov-Smirnov normality test was first performed. The independent *t* test was used to compare data between the groups, and the dependent *t* test was used to perform intragroup comparisons. Correlations between continuous variables were made using the Pearson correlation test or Spearman correlation test. The statistical significance level was set at 5% for all tests (*p* < 0.05). Statistical analysis was performed using statistical software (Statistical Package for Social Sciences 19.0®, Chicago, IL, USA).

### Sample Size

The calculation of the sample size was based on a previous study by Pardina et al. [31] that identified the plasma parameters of obese patients before and at 1, 6, and 12 months after BS as the outcome. Average values of leptin (ng/mL) were used to calculate the sample size of 30 subjects, which was determined with an α error of 0.05 and a β error of 95% power to detect a high effect size of 0.88.

## Results

### Effect of Weight Loss Induced by BS on Anthropometric Parameters

The main anthropometric, demographic, and clinical characteristics of patients in the CG and BSG are shown in Table 1. All patients were classified as being morbidly obese at baseline assessment, with a mean BMI of 47.5 ± 5.6 kg/m^2^ in the CG and BMI of 47.1 ± 6.3 kg/m^2^ in the BSG. In the baseline evaluation, no significant difference was observed between the CG and BSG in sex, age, weight, BMI, systolic blood pressure (SBP), and diastolic blood pressure (DBP), demonstrating homogeneity of the sample. The same result was also observed when we compared the baseline variables and those at 6 months of follow-up in the CG. However, weight, BMI, SBP, and DBP at baseline and the 6-month follow-up were significantly different in the BSG.

**Table 1:** Anthropometric, demographic and clinical characteristics of the subjects involved in the study

Variables	CG T1 (*n* = 14)	CG T2 (*n* = 14)	*p* value	BSG T1 (*n* = 55)	BSG T2 (*n* = 55)	*p* value
Gender (%)						
Female	78.5	78.5		90.9	90.9	
Male	21.4	21.4		9.0	9.0	
Age	40.7 ± 11.8	40.7 ± 11.8		41.8 ± 9.6	41.8 ± 9.6	
Weight (kg)	125 ± 29	126.8 ± 29.1	ns	126.1 ± 19.7	88.3 ± 15	***
Height (cm)	161 ± 0.1	161 ± 0.1		161 ± 0.1	161 ± 0.1	
BMI (kg/cm^2^)	47.5 ± 5.6	48 ± 5.1	ns	47.1 ± 6.3	33 ± 5.5	***
Ethnicity (%)						
Caucasoid	71.5	71.5		85.4	85.4	
Negroid	28.5	28.5		14.6	14.6	
SBP	131 ± 11.4	132.3 ± 15.1	ns	128.8 ± 17.8	119.3 ± 6.8	***
DBP	88.7 ± 7.4	88.8 ± 8.9	ns	86.9 ± 14.9	77.1 ± 4.4	***

CG control group, BSG bariatric surgery group, T1 time 1, T2 time 2, SBP systolic blood pressure, DBP diastolic blood pressure, ***p < 0.005

### Effect of Weight Loss Induced by BS on Lipid Metabolism and Inflammatory Blood Markers

The lipid metabolism variables, glucose, TC, HDL cholesterol, LDL cholesterol, triglyceride, and circulating concentrations of inflammatory markers, TNF-α, adiponectin, and leptin, are shown in Table 2. There were no significant differences when comparing all variables in T1 and T2 for CG. There was a slight increase in the mean value of the variables, except for adiponectin that showed a slight decrease. We observed a significant difference between all lipid and biochemical metabolic variables in the blood samples obtained preoperatively and at 6 months postoperatively in patients in the BSG. We also observed this result for the circulating inflammatory markers, highlighting the considerable increase in values relating to adiponectin.

**Table 2:** Biochemistry and inflammatory blood markers of the subjects involved in the study

Variables	CG T1 (*n* = 14)	CG T2 (*n* = 14)	*p* value	BSG T1 (*n* = 55)	BSG T2 (*n* = 55)	*p* value
Glucose	97.5 ± 12	103.1 ± 9	ns	103.5 ± 12.5	86.4 ± 8	***
Total cholesterol	197 ± 21.5	201.2 ± 24.1	ns	197.2 ± 33	120.2 ± 21.1	***
HDL	41 ± 6.9	43.4 ± 6.2	ns	47 ± 12.7	52.7 ± 10.1	***
LDL	122.8 ± 36.9	126 ± 25.9	ns	132.6 ± 28.9	96.2 ± 20.9	***
Triglycerides	122.6 ± 39.8	125 ± 38.6	ns	153.9 ± 55.2	89.6 ± 19.7	***
TNF-α	0.9 ± 0.1	1.2 ± 0.4	ns	0.8 ± 0.3	0.2 ± 0.2	***
Adiponectin	0.6 ± 0.1	0.4 ± 0.1	ns	0.3 ± 0.3	1.3 ± 0.6	**
Leptin	1.7 ± 0.5	2 ± 0.5	ns	1.9 ± 0.5	0.4 ± 0.4	***

CG control group, BSG bariatric surgery group, T1 time 1, T2 time 2, HDL high-density lipoprotein, LDL low-density lipoprotein, TNF-α tumor necrosis factor, **p < 0.05, ***p < 0.005

**Fig. 2:**
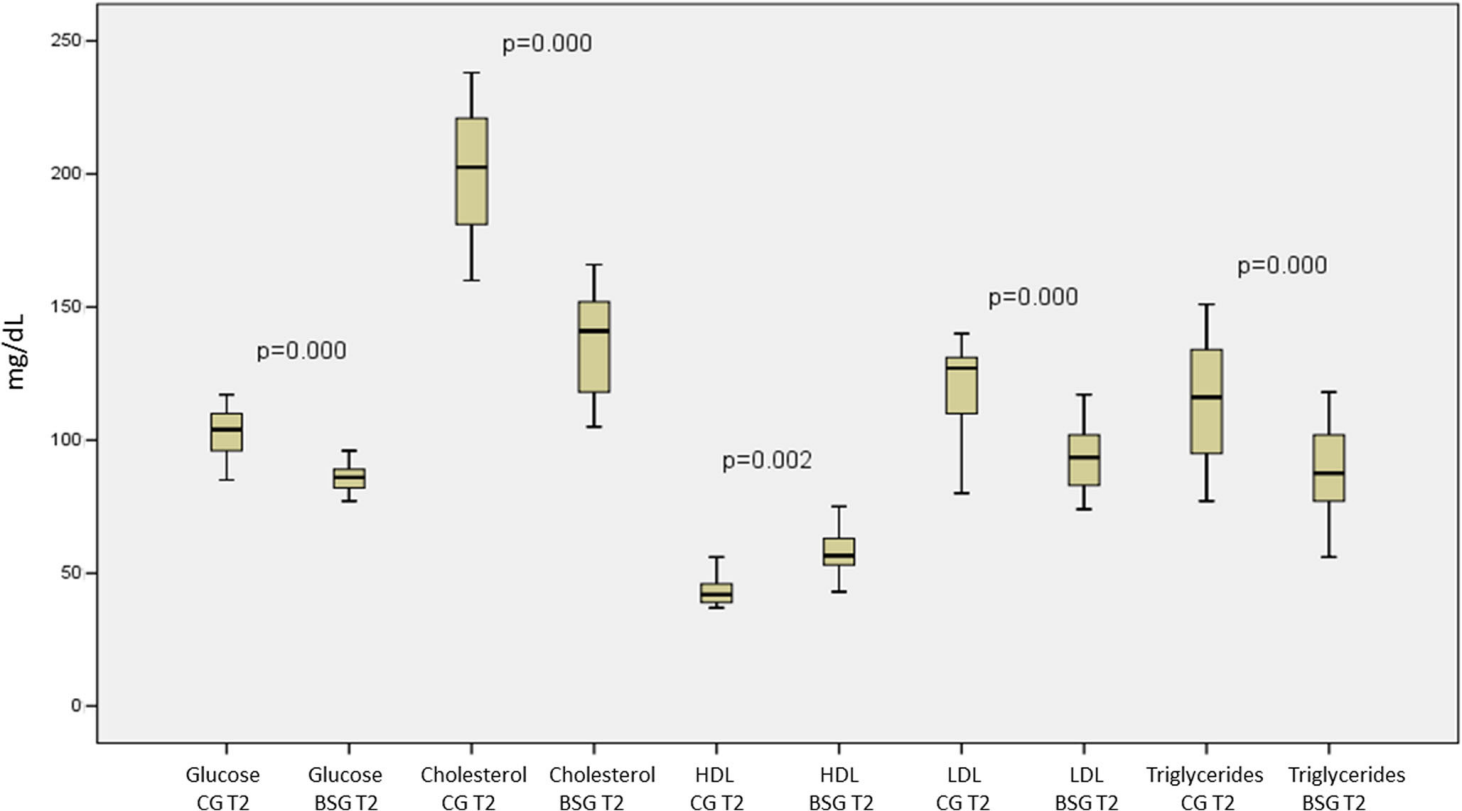
Biochemical blood variables of control group vs. bariatric surgery group in the follow-up evaluation. BSG bariatric surgery group, CG control group, T2 time 2, HDL high-density lipoprotein cholesterol, LDL low-density lipoprotein cholesterol

**Fig. 3:**
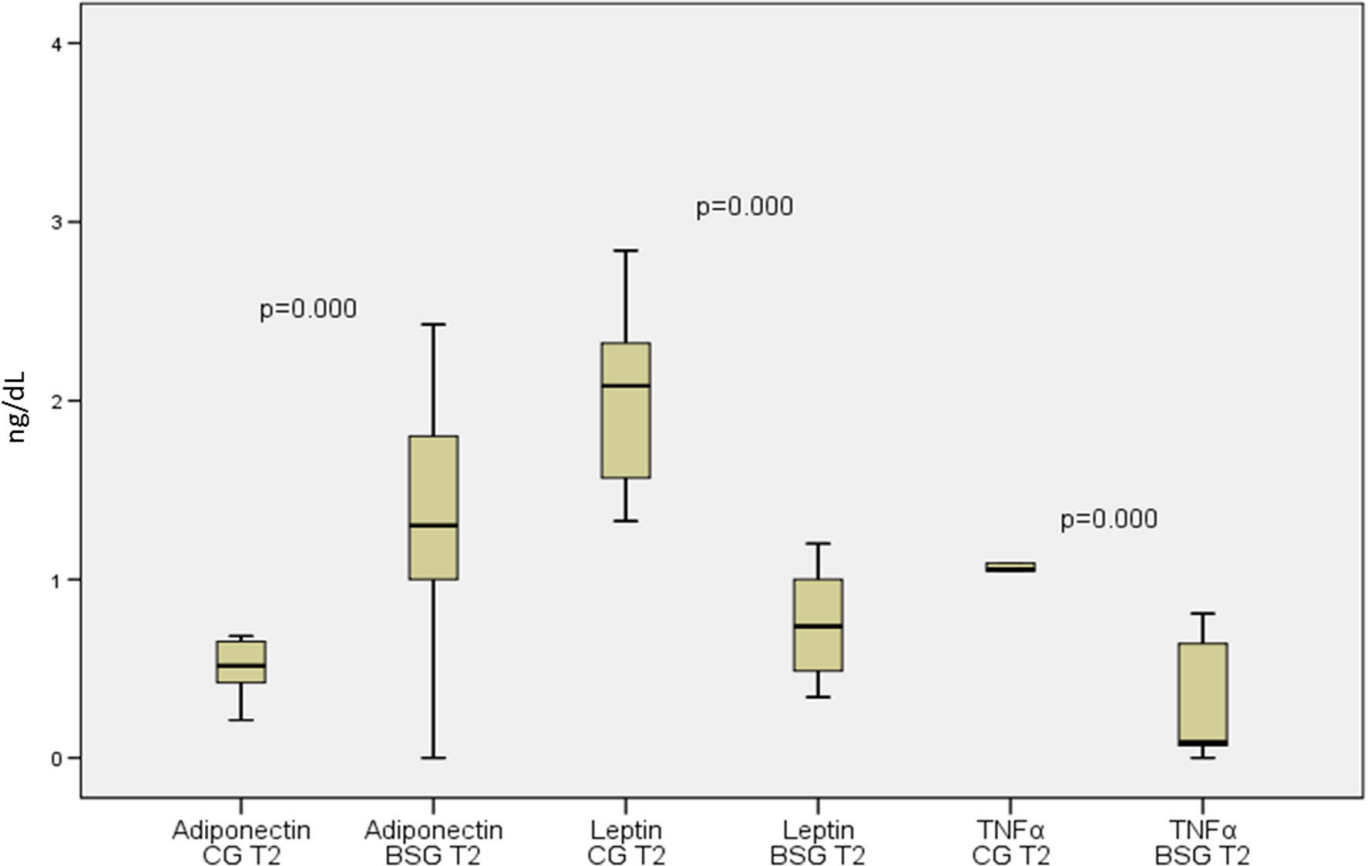
Inflammatory markers of control group vs. bariatric surgery group in the follow-up evaluation. CG control group, BSG bariatric surgery group, TNF-α tumor necrosis factor α

**Fig. 4:**
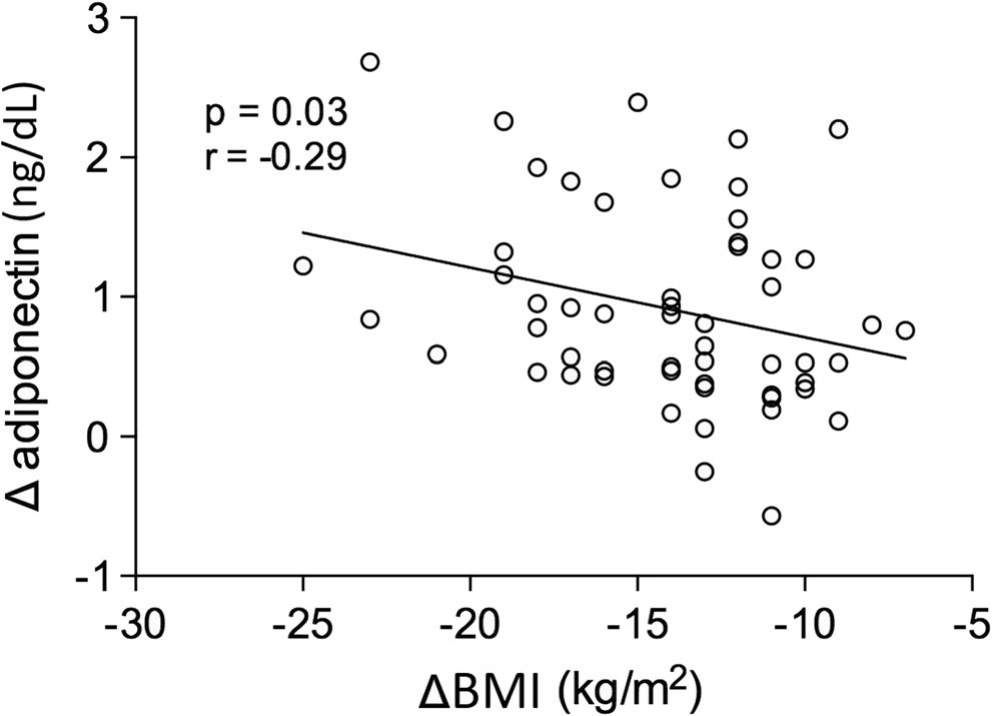
Correlation between the delta of the body mass index and the serum adiponectin delta of patients in the bariatric surgery group. ng/dL nanogram per deciliter, kg/m^2^ kilogram per square meter, Δ delta, BMI body mass index

**Fig. 5:**
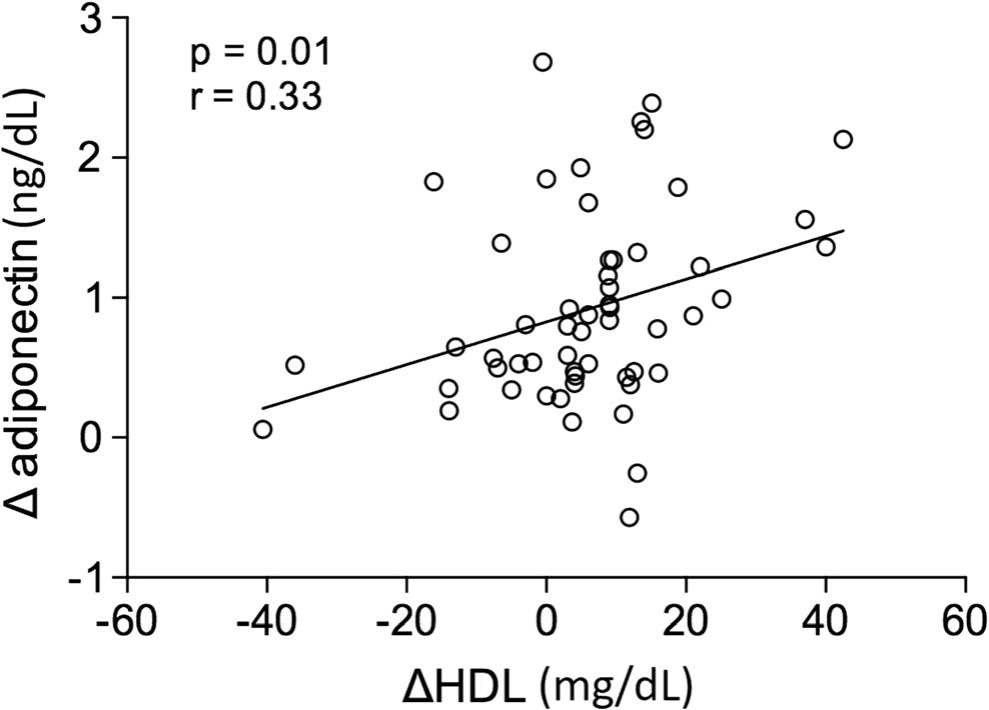
Correlation between the high-density cholesterol delta and the serum adiponectin delta of patients in the bariatric surgery group. ng/dL nanogram per deciliter, mg/dL milligrams per deciliter, Δ HDL delta high-density cholesterol

Figure 2 shows changes in the metabolic and biochemical variables of patients in the CG versus the BSG at the second evaluation 6 months after the baseline evaluation. All variables analyzed were significantly different between the two groups. It should be noted that no graphical representation was made to compare the same baseline variables between these groups because no significant difference was observed. Figure 3 shows the same result for the inflammatory markers. Adiponectin values were significantly increased, and leptin and TNF-α were significantly decreased when we compared the CG with the BSG at the 6-month follow-up. In Fig. 4 we can observe a positive correlation (Spearman) between the BMI delta and the adiponectin delta, with a value of *p* < 0.03. A positive correlation (Spearman) was also observed between adiponectin and HDL cholesterol levels with a value of *p* < 0.01 (Fig. 5).

## Discussion

Recently, obesity has been characterized by a low-grade inflammatory state known as inflammome, indicated by chronic increases in circulating concentrations of inflammatory markers, including pro-inflammatory cytokines (e.g., IL-6 and TNF-α) and protein C [27, 32]. It is believed that this inflammatory condition is due to the presence of large amounts of adipose tissue, which is consistent with studies that demonstrated the association between circulating inflammatory markers and adipose tissue with variables of central adiposity [33, 34].

We chose to analyze leptin because it is a pro-inflammatory cytokine secreted by adipose cells from subcutaneous cellular tissue and it increases considerably in obese patients. Adiponectin is an anti-inflammatory adipokine secreted by subcutaneous cellular tissue, and TNF-α is not produced by subcutaneous cellular tissue in vivo [35–37]. Samples of adipose tissue from an obese person have an infiltration of macrophages, which explains the large number of secreted pro-inflammatory mediators. This finding confirms that the chronic inflammatory state is related to obesity [22, 38]. TNF-α is secreted by macrophages presented on stromal vascular tissue from adipose tissue and represents a pro-inflammatory adipokine, providing a central role in insulin resistance, causing the phosphorylation of the substrate-1 of the insulin receptor, and avoiding its pairing [39]. Leptin an inflammatory marker and marker of long-term accumulated body energy, and its relationship with other inflammatory markers has no link with BMI [40]. Some studies have shown a close relationship between leptin and TNF-α once it induces the production of leptin [41], and leptin induces the production of TNF-α from macrophages [42].

The relationship between adiponectin and TNF-α is not completely clear [39]. Mallipedhi et al. published a study analyzing inflammatory markers after sleeve gastrectomy, and they observed a decrease of 43% in leptin 6 months postoperatively [43]. Additionally, they showed that adiponectin increased and tended to be better, but this did not reach statistical value.

Another study showed an improvement of the inflammatory profile of obese patients who consumed a low-calorie diet and had a weight loss of 9% after 21 days, but it did show any important decrease in TNF-α [44]. Additionally, the authors suggested removing fatty tissue to reach a better result of TNF-α.

Our study showed statistical significance in all three markers studied. When we compared the CG with the BSG, we found some interesting results. BS improved values of all three inflammatory markers 6 months postoperatively. However, in the CG, we found a worsening TNF-α level, meaning that in the CG, patients did improve (*p* < 0.05). This result did not occur with adiponectin and leptin, which were stable in the CG 6 months later after no surgery.

Sams et al. investigated the effects of laparoscopic Roux-en-Y gastric bypass and laparoscopic gastric band on serum and tissue levels of adiponectin and serum levels of monocyte chemotactic protein-1 and TNF-α in a sample of 20 patients [22]. The authors observed a significant increase in the values of adiponectin compared to the values at baseline and 2 weeks and 6 months postoperatively. This result was similar to that observed in our study.

Ibrahim et al., in 2017, showed the association of some inflammatory markers with cardiovascular risk and showed that adiponectin has an inverse relation to the risk of coronary stenosis [45]. In our study, a variation of adiponectin in the postoperative period was observed from 0.293 to 1.2 ng/dl, which corroborates with the aforementioned study and the consequent reduction of cardiovascular risk in the patients studied.

Another important result of our study was the negative correlation of the delta values of adiponectin with the delta of the BMI. As these markers improved their values after BS, we can infer that the inflammatory response reduced, which may lead to a decrease in the association between obesity and cardiovascular risk and cancers, especially those related to obesity such as endometrial cancer [46].

According to Christou et al., adiponectin has a direct correlation with HDL and inversely proportional to triglycerides. The elevation of adiponectin following bariatric surgery may also explain the results obtained with altered HDL and triglycerides [47]. In our study, a positive correlation (Spearman) was observed between the values of adiponectin and HDL. A negative correlation (Spearman) was also observed between the values of adiponectin and triglycerides, but not significant. These results corroborate with the study by Christou et al.

Our findings also corroborate the study by Arismendi et al. [27] that showed for the first time that systemic inflammome is associated with morbid obesity. The authors included 129 patients (96 women) with a mean age of 43 ± 7 years who underwent BS and 20 healthy controls. Sleeve gastrectomy was performed in 68 (53%) obese patients, and Roux-en-Y gastric bypass was performed in 61 (47%). Patients undergoing BS were evaluated at baseline and 1 year postoperatively. They observed a significant reduction in serum concentration levels of leptin and TNF-α and an increase in adiponectin values. These results confirm that morbid obesity is associated with a remarkable systemic inflammation component, indicating that obesity per se is likely the main driving force of systemic inflammation in this clinical setting.

As these markers improved after BS, we can deduce that the inflammatory response is better, and this may lower the incidence of some cancers among obese people. In other words, we can deduce that BS may lead to a decrease in the incidence of cancers, especially those related to obesity. Another interesting point of our analyses is that the presence of diabetes did not affect the postoperative results, if compared with the postoperative results of non-diabetic patients. The same finding was reported by Thomsen et al. [48].

With this study, we can infer that severe obese patients who have not achieved a considerable weight reduction and maintained through clinical methods should be referred for surgical treatment. However, we agree with Ray et al. (2017), who advocate the need for longitudinal studies to elucidate the causal relationship of obesity and inflammatory markers or otherwise, as well as the need for randomized controlled multicenter trials to confirm the beneficial effect of bariatric surgery on the inflammatory state of obese patients [49].

We would like to emphasize that it would be very important to follow these patients for a longer period of 1–2 years in order to verify the behavior of the inflammatory profile and its relation with the comorbidities presented by the patients at the beginning of the study. We would also like to draw the attention of the scientific community to the importance of analyzing other pro-inflammatory and anti-inflammatory markers in samples of adipose tissue and blood from severe obese patients undergoing bariatric surgery.

### Study Limitations

One of the limitations of our study was the small number of male patients, which could alter the results of the analyzed variables because of the constitutional difference of lean mass, distribution of adipose tissue, and differences in food intake. However, despite the large number of patients in the BSG who withdrew from the study without completing the 6-month follow-up, the study sample exceeded the sample calculation required. We believe that it will be very important to follow-up with these patients again for 1 year after surgery to verify our findings.

## Conclusions

Our study showed that 6 months after BS, there was significant improvement in relation to the blood metabolic and biochemical variables and an increase in adiponectin values, which was associated with a decrease in leptin and TNF-α levels in severely obese patients. Therefore, weight loss induced by BS reduced the inflammome state in severely obese patients. We believe that new studies with a large number of patients and long-term follow-up are necessary to reinforce our findings.
